# Auditing fairness in clinical AI systems using provenance-based simulation: a comparative and regulatory perspective

**DOI:** 10.3389/frai.2026.1756023

**Published:** 2026-04-10

**Authors:** Fidelis Alu, Sunkanmi Oluwadare, Nnennaya Ngwanma Halliday, Uchenna Emmanuel Agwunobi

**Affiliations:** 1 School of Information Technology, University of Cincinnati, Cincinnati, OH, United States; 2 University of Texas, Southwestern Medical Center, Dallas, TX, United States

**Keywords:** algorithmic fairness, bias detection, clinical AI, explainable AI, model governance, provenance auditability

## Abstract

**Introduction:**

The adoption of Artificial Intelligence (AI) in clinical decision support has encountered obstacles due to algorithmic bias and lack of transparency. To address this, we developed an auditing framework using detailed data provenance to audit fairness.

**Methods:**

We simulated audits on a synthetic patient dataset (*N* = 1,000), comparing logistic regression and random forest models to detect gender bias using 5-fold crossvalidation and permutation testing.

**Results:**

Logistic regression achieved 75.2 ± 1.0% accuracy (AUC = 0.806 ± 0.030) and random forest achieved 70.1 ± 1.4% accuracy (AUC = 0.745 ± 0.020). Provenance logs successfully detected gender biases in both models. Logistic regression exhibited statistically significant bias (EOD = +0.256, *p* = 0.0080), while random forest’s smaller disparity (EOD = +0.055, *p* = 0.5664) was not statistically significant, demonstrating that our audit distinguishes systematic discrimination from random variation. Sensitivity analysis confirmed successful bias detection across magnitudes from *β* = −0.10 to *β* =−0.80.

**Discussion:**

Despite lower accuracy, random forest showed 57% less bias than logistic regression, challenging assumptions that interpretability guarantees fairness. We introduce a standardized AI Fairness Provenance Record documenting data origin, model choices, and bias metrics, enabling auditors to trace decisions to their source. This framework maps to FDA transparency guidelines and ONC HTI-1 requirements, demonstrating how provenance-based auditing supports regulatory compliance and provides a pathway toward more responsible and equitable AI in clinical settings.

## 1 Introduction

Healthcare systems have quickly incorporated AI tools for tasks like risk assessment and diagnosis. These tools have demonstrated promise in enhancing workflow efficiency and patient outcomes (Obermeyer et al., 2019; Li et al., 2023). However, there is growing concern that biases in healthcare data may be unintentionally reinforced or even amplified by the use of clinical AI. One prominent example is the hospital risk algorithm examined by Obermeyer et al. (2019), which consistently underestimated Black patients’ needs by using healthcare costs as a stand-in for health needs, a faulty metric reflecting past disparities in access. The biased algorithm systematically underestimated risk for Black patients, denying them access to high-risk care programs at the same rate as equally sick white patients (Obermeyer et al., 2019). Correcting this bias would have increased the number of Black patients receiving extra care from 17.7 to 46.5% (Obermeyer et al., 2019). This case is not an anomaly. Systematic review studies, like that by Li et al. (2023), consistently showed that models for cardiovascular risk and other conditions demonstrated uneven performance across race and gender groups, with mitigation efforts often proving ineffective when there are biases woven into correlated variables. This aligns with the broader AI fairness literature: Fairness can be measured in many ways (e.g., demographic parity, equalized odds, disparate impact), but achieving it requires data and model behavior throughout the AI lifecycle to be carefully scrutinized. These examples show that *algorithmic fairness is not guaranteed*: models can reproduce historical inequalities unless actively audited and corrected. If left unattended, they will lead to clear consequences such as the erosion of trust, misallocation of resources, misdiagnosis, and inequitable healthcare outcomes (Chinta et al., 2025). A recent thesis by Alu (2023) further cautioned that if AI bias in high-stakes systems is not addressed, it could lead to reputational damage, discrimination, and even loss of life.

A major challenge in addressing this issue is the “black box” problem. For clinicians and regulators, the internal logic of complex models remains opaque, making it nearly impossible to answer very critical questions: Was a patient’s gender a decisive factor? Was the training data representative enough? While external audits can sometimes diagnose symptoms, they often miss the root cause because the algorithm’s decision pathway is hidden. Researchers have resorted to external audit studies and clever workarounds to diagnose biases from the outside (Obermeyer et al., 2019). However, these *ad hoc* audits are labor-intensive and may miss root causes because the actual algorithm logic remains hidden. What if, instead, AI systems were designed to be *auditable by default*? Recent expert panels and regulators have called for exactly this: to establish more policies in deploying AI in the healthcare domain to ensure they are fair, equitable, and explainable (Hasanzadeh et al., 2025). Therefore, AI-enabled clinical decision support should come with documentation and logging that reveal how data influence outcomes. This push has so far led to a growing consensus among experts and regulators: for AI to be trusted in clinical settings, it must be auditable by design (Hasanzadeh et al., 2025). Amann et al. (2022) noted that explainability is only one facet of transparency, it is equally important to have detailed documentation of data sources, model development, and their known limitations. In other words, to trust an AI, one must be able to inspect its provenance: the origin of its inputs and the path of its decision process. Recent work in clinical AI auditing has begun to operationalize this vision. Barnard et al. (2024) proposed a responsible MLOps design methodology for auditing AI-based clinical decision support systems, emphasizing lifecycle governance from development to post-deployment. Barnard et al. (2023) introduced MACAIF, a framework specifically targeting machine learning auditing for clinical AI fairness. Menéndez (2022) explored measuring ML robustness in adversarial settings, highlighting the need for systematic evaluation beyond standard performance metrics. Our work builds on and extends these contributions by embedding provenance tracking directly into the audit process and mapping the resulting framework to specific regulatory requirements.

This paper presents a methodology for making clinical AI systems auditable by embedding provenance tracking from the outset. We define provenance as the complete historical record of data and the decision processes applied to it. In our simulation, we trained two common models, a transparent logistic regression and a more complex random forest, on a synthetic clinical dataset with a deliberately embedded gender bias. By maintaining provenance logs, essentially an “audit trail” of each model decision, we enable systematic bias checking that is otherwise impractical. We conduct a simulation study on a synthetic clinical dataset to illustrate how such logs can facilitate a fairness audit. Two prediction models (a logistic regression and a random forest) are trained to predict 30-day hospital readmission, using gender as the sensitive attribute. The dataset was constructed with a built-in gender coefficient (*β* = −0.30) to examine whether and how each model learns and propagates that bias. Using *provenance logs*, we trace each model’s decision logic and measure fairness metrics such as demographic parity and Equal Opportunity Differences (Amann et al., 2022).

To ensure methodological rigor, we employed 5-fold stratified cross-validation with 95% confidence intervals and permutation-based statistical significance testing. We additionally conducted sensitivity analysis across bias magnitudes from *β* = −0.10 (subtle) to *β* = −0.80 (extreme) to evaluate audit robustness across varying levels of discrimination.

Our work makes five key contributions:

1. *A practical, statistically validated audit framework*: We move from theory to practice by demonstrating a simulated fairness audit powered by provenance logs, validated through 5-fold cross-validation and permutation significance testing. This framework shows how every step of an AI system’s reasoning can be recorded, and bias detection claims can be distinguished from random variation, a critical requirement for trustworthy auditing.
2. *A comparative analysis of bias mechanisms with a counterintuitive finding*: We go beyond simply noting bias to revealing its different pathways across model types. By comparing logistic regression and random forest, we demonstrate that while both models can propagate bias, the mechanisms differ significantly. Critically, our results reveal that the interpretable logistic regression exhibited stronger, statistically significant bias (EOD = +0.256, *p* = 0.0080) compared to the “black box” random forest (EOD = +0.055, *p* = 0.5664). This counterintuitive finding that interpretability does not guarantee fairness, challenges a common assumption in the clinical AI community and is a key empirical contribution of this work.
3. *Audit sensitivity across bias magnitudes*: Through systematic sensitivity analysis, we demonstrate that our provenance-based audit successfully detects gender bias across magnitudes ranging from subtle (*β* = −0.10, EOD = +0.158) to extreme (*β* = −0.80, EOD = +0.298). This robustness to varying bias levels is essential for real-world deployment, where the magnitude of potential discrimination is unknown *a priori*.
4. *The AI fairness provenance record (AFPR)*: We propose and prototype a standardized, structured log for recording fairness-critical information. This record captures data lineage, model version history, per-decision audit trails, and bias metrics, providing hospitals with a tangible tool for ongoing AI governance and accountability. The AFPR is implemented as a structured JSON schema with complete documentation, enabling practical adoption by healthcare institutions.
5. *Regulatory compliance mapping*: We directly map our provenance-based approach to emerging regulatory requirements, demonstrating how the AFPR provides specific documentation supporting compliance with the U.S. FDA’s transparency guidelines for machine learning-enabled medical devices (U.S. Food and Drug Administration, 2024) and the algorithmic transparency mandates of the ONC HTI-1 rule (Dlapiper, 2023). By providing a clear pathway from technical audit to regulatory compliance, this work addresses a gap between technical fairness research and practical implementation in regulated healthcare environments.

In essence, we argue that preemptive auditability is the most viable path toward trustworthy clinical AI. By making bias traceable, our provenance-driven approach provides the evidence base needed for hospitals to certify fairness and for regulators to hold systems accountable. The following sections detail our methodology and findings, building a case for why provenance is not just a technical feature, but a foundational component of ethical AI in healthcare.

## 2 Related work

The problem of algorithmic bias in healthcare is now well-established in several literature, and has become a major research focus in recent years. Bias in this context refers to systematic performance disparities between patient groups (e.g., across race, gender, age) that lead to unfair or harmful outcomes (Hasanzadeh et al., 2025). Research has so far moved from just initial case studies documenting disparities to more complex arguments surrounding how to define and measure fairness in clinical settings. A variety of fairness metrics have been proposed: *demographic parity* requires equal positive prediction rates across groups, *equalized odds* requires equal true positive and false positive rates (Hardt et al., 2016), and *equal opportunity* requires equal true positive rates for those who qualify for the outcome. Applying standard fairness metrics such as demographic parity or equalized odds (Hardt et al., 2016), is not straightforward. In healthcare, these definitions must be applied carefully because patient populations and error costs differ by context, and failing to apply this can lead to negative consequences (Chen et al., 2023). A critical insight from Obermeyer et al. is that the training data itself can be a source of bias. Their analysis revealed that an algorithm perpetuated racial bias because it relied on healthcare costs as a proxy for health status, a metric shaped by long-standing inequities in access to care (Obermeyer et al., 2019). This demonstrates that effective bias mitigation requires checking of the entire modeling pipeline, from the initial data labels to its final output.

Studies have documented bias in numerous clinical algorithms. Beyond the risk score example, researchers have found racial biases in pulse oximeters and clinical risk calculators, gender biases in outcomes prediction for cardiovascular disease (Fawzy et al., 2022), and algorithmic heterogeneity in sepsis risk stratification (Fohner et al., 2019). A systematic review by Kumar et al. (2023) reported that over half of published healthcare Machine Learning (ML) models had high risk of bias due to non-representative data or poor validation practices. Only a small fraction adequately addressed bias or performed external validation on diverse cohorts. These findings highlight an urgent need for standardized bias auditing practices (Kumar et al., 2023).

### 2.1 Existing fairness tools and their limitations

In response to documented bias the machine learning community has developed software tools designed to quantify and mitigate bias. Toolkits such as IBM’s *AI Fairness 360* (Bellamy et al., 2019) and Microsoft’s *FairLearn* (Bird et al., 2020) provide implementations of common fairness metrics and algorithms for bias mitigation (Pagano et al., 2023). These tools can compute statistics like disparate impact (ratio of positive rates between groups) and suggest interventions (rebalancing training data, algorithmic adjustments, etc.). While useful, these tools typically operate as external audits. They generate a static assessment at a single point in time, dependent on the developer to initiate the analysis. They do not integrate into the live system’s ongoing operation and cannot alert practitioners to emerging biases in real time. Concurrently, the proposal of *Model cards* are concise reports accompanying ML models that detail their intended use, performance across subgroups, and evaluation conditions (Mitchell et al., 2019). The goal is to surface any ethical considerations, including bias, to stakeholders in a standardized way. Likewise, the *datasheets for datasets* communication by Gebru et al. (2021) have been proposed to document how training data was collected, its demographic makeup, and potential sources of bias, so that downstream users are aware of limitations. These initiatives mirror the disclosure requirements common in other regulated sectors.

While model cards and fairness toolkits provide valuable frameworks, they share a fundamental limitation: they are static documents or one-time analyses, separate from the AI system’s ongoing operation. What’s missing is integration into the *live* system. Our work extends these ideas by embedding fairness checks into the AI’s ongoing workflow via provenance logging. In effect, we aim to automate a continuously updating “model card” in the form of a provenance record that logs how each decision was made and how the model is performing for different groups in real time. This dynamic audit trail complements static documentation and can alert practitioners (whether physicians or healthcare administrators) to emerging biases (for instance, if a model’s accuracy starts to drop for a certain subgroup over time).

Critically, our framework is designed to be complementary to, not competing with, existing tools such as FairLearn and AI Fairness 360. Those tools provide mitigation algorithms; our framework provides the continuous audit trail needed to detect bias and guide selection of appropriate interventions. For example, if provenance logs reveal that a protected attribute carries high feature importance, FairLearn’s reweighting approach may be an appropriate response. If logs reveal that bias operates through proxy features correlated with gender or race, adversarial debiasing strategies may be more suitable. Provenance auditing thus serves as the diagnostic layer that informs and directs mitigation.

### 2.2 Clinical AI auditing frameworks

The concept of auditable AI systems has been explored in related contexts such as finance and security, but is only recently gained traction in healthcare. Labkoff et al. (2024) recommended that AI-based clinical decision support tools should come with robust validation, continuous monitoring, and transparency for users. They envision lifecycle governance, from development to post-deployment, to ensure safety and effectiveness (Labkoff et al., 2024). Recent work has begun to operationalize this vision with specific frameworks. Barnard et al. (2024) proposed a responsible MLOps design methodology for auditing AI-based clinical decision support systems, providing structured guidance on integrating auditing into model development and deployment pipelines. Barnard et al. (2023) introduced MACAIF (Machine Learning Auditing for Clinical AI Fairness) a framework specifically targeting fairness evaluation in clinical ML systems, demonstrating the feasibility of structured fairness auditing in healthcare contexts. Menéndez (2022) explored the measurement of ML robustness in adversarial settings, highlighting the importance of systematic evaluation approaches that go beyond standard performance metrics. Our work builds directly on these contributions by adding provenance-based tracking as the technical mechanism for continuous audit, and by providing explicit mapping to current regulatory requirements, two elements not addressed by prior frameworks.

Provenance logging is a major enabler to actualizing this vision. Ahmed et al. (2023) define data provenance in healthcare as “attributes about the origin of health information at the time it is first created and track the uses and permutations of the health information over its lifecycle”. Provenance systems can utilize detailed logs, cryptographic hashes, or even blockchain to ensure that every action on data is traceable and tamper-proof (Zubair, 2025). For example, every time our AI model reads a patient record or issues a recommendation, a secure log entry can record what happened. Prior work suggests that such *immutable audit logs* provide accountability and make it feasible to retrospectively investigate errors or biases (Joseph, 2023). Our framework builds on these ideas, applying them explicitly to fairness auditing. By combining explainable AI techniques with secure provenance tracking, we strive to meet the dual requirement highlighted by regulators and clinicians: AI systems should be both *transparent in evidence and fully auditable in operation*.

### 2.3 Regulatory landscape

The push for AI fairness and transparency is reinforced by developments within regulatory bodies. Like the European Commission, U.S. FDA, Health Canada, and WHO have all increased their efforts toward more strict AI governance, emphasizing fairness, equity, and explainability (World Health Organization, 2023a,b; Silva et al., 2022; Gichoya et al., 2023; Directorate-General for Parliamentary Research Services (European Parliament) et al., 2022; Mennella et al., 2024). In 2021 The FDA issued guiding principles for Good Machine Learning Practice, as well as joint publication on *Transparency Guidelines for ML Medical Devices* with other agencies. These emphasize clear communication of a device’s intended use, logic (how it makes decisions), and known limitations or biases (Mennella et al., 2024). For example, the FDA guidance suggests that developers should document and disclose any known biases or gaps in the data used, such as underrepresentation of certain demographic or population (Mennella et al., 2024). In the United States healthcare system, a significant recent development is the *ONC’s HTI-1 rule (2023)*, which establishes algorithmic transparency requirements for clinical software. This regulation requires that certified health IT products must provide users (clinicians) with information on the algorithm’s input features (including if demographic or social factors are used), its intended scope, performance, and importantly any fairness or bias mitigation measures taken (Dlapiper, 2023). Developers must also supply quantitative measures of fairness (e.g., validation performance stratified by demographics) and a schedule for ongoing bias monitoring (Dlapiper, 2023).

Our proposed AI Fairness Provenance Record (AFPR) directly addresses these regulatory requirements. By design, it records input data details, model behavior, per-decision audit trails, and bias metrics, the precise information these guidelines mandate. In the sections that follow, we detail how we implemented the provenance-based audit in a simulated study, what our experiments demonstrated, and how this approach supports both practical healthcare AI governance and regulatory compliance.

## 3 Methodology

Our methodology involves creating a controlled simulation in which we can inject a known bias into data, apply two different machine learning (ML) models, and then audit their behavior using provenance logs. The goal is to emulate how a fairness audit might be conducted on a hospital’s AI system in practice, except in our case, we know the “ground truth” about the bias and can validate whether the audit correctly detects it. The two model types, *logistic regression* and *random forest*, were chosen for their contrasting properties: logistic regression is a simple, linear model whose decisions are relatively transparent (weights on features), whereas random forest is a more complex, non-linear ensemble that can be considered a “black box” without additional interpretation tools. By comparing them, we were able to illustrate the challenges of bias detection across model types and examine whether model interpretability is related to model fairness, a question with direct implications for clinical AI deployment decisions.

### 3.1 Research hypotheses

Prior to conducting the audit, we formulated the following hypotheses:

*H1*: Both models would learn the gender bias present in the training data, resulting in positive Demographic Parity Difference (DPD) and Equal Opportunity Difference (EOD) values indicating systematic differences in prediction patterns between male and female patients.

*H2*: The logistic regression model would exhibit stronger, more systematic bias than the random forest, due to its direct linear encoding of gender as a weighted feature, a mechanism that applies a uniform penalty to one group across all predictions.

*H3*: The provenance-based audit would successfully detect and characterize gender bias across varying magnitudes of discrimination, from subtle (*β* = −0.10) to extreme (*β* = −0.80), demonstrating robustness for real-world deployment where bias magnitude is unknown a priori.

### 3.2 Synthetic clinical dataset

We created a synthetic dataset of 1,000 patient records for a 30-day hospital readmission prediction task. Each record contained four clinical features: age (years, range 20–85), body mass index (BMI, normally distributed with mean 27 and standard deviation 5, clipped to range 16–45), comorbidity status (binary, 35% prevalence), and gender (binary, 0 = female, 1 = male, approximately 50% each).

The binary readmission outcome was generated using a logistic function with the following coefficients:

- Intercept: −1.0
- Age (centered and scaled): *β* = +0.50
- BMI (centered and scaled): *β* = +0.40
- Comorbidity: *β* = +1.00
- Gender (male = 1): *β* = −0.30

The gender coefficient (*β* = −0.30) assigns male patients a systematically lower probability of readmission compared to equally sick female patients. This coefficient was selected based on the following considerations. First, a magnitude of 0.30 on the log-odds scale represents a clinically meaningful but not extreme disparity, comparable in magnitude to documented biases in published clinical risk algorithms (Obermeyer et al., 2019). Second, this value produces clearly detectable but not overwhelming fairness disparities (EOD ≈ 0.25), enabling demonstration of audit sensitivity without creating a trivially obvious bias. Third, our sensitivity analysis (Section 3.4.4) validated this choice by demonstrating that the audit framework successfully detects bias across a full range of magnitudes from *β* = −0.10 to *β* = −0.80, confirming that our main experiment represents a realistic and representative scenario.

### 3.3 Justification for synthetic data and simplified feature space

We deliberately used synthetic data with a limited feature set (four features) for three methodological reasons. First, synthetic data with a known injected bias provides the ground truth necessary for audit validation. In real clinical data, the true magnitude and nature of bias are unknown, making it impossible to confirm whether an audit correctly identified all sources of discrimination. Our controlled design allows definitive validation: we can confirm that the audit detects precisely the bias we deliberately introduced. Second, the simplified feature space isolates the audit mechanism from confounding factors present in high-dimensional real-world data, multiple simultaneous biases, missing data patterns, measurement errors, and complex feature interactions, that would complicate attribution of detected disparities to specific sources. Third, this approach follows established practice in methods validation research, analogous to unit testing in software engineering: test individual components in controlled settings before integration testing in complex environments. The limitations of synthetic data and the path toward real-world validation are discussed in Section 4.4.

All analyses were conducted in Python using scikit-learn, with random seed set to 42 for reproducibility. The complete dataset and analysis code are publicly available (see Data Availability Statement).

### 3.4 Model training

The dataset was divided into training and test sets using a stratified 70/30 split (700 training, 300 test patients), with stratification by the outcome variable to preserve class balance across splits. Both models were trained on identical training data and evaluated on the same held-out test set.

*Logistic regression* was trained using the liblinear solver with a maximum of 1,000 iterations. The model takes the form: ŷ = *σ*(w₀ + w₁·age + w₂·bmi + w₃·comorbidity + w_gender·gender), where σ is the sigmoid function. This formulation captures bias by assigning a coefficient w_gender to the sensitive attribute, directly encoding any learned gender disparity as a scalar weight applied uniformly across all predictions.

*Random forest* was trained with 100 estimators using default scikit-learn parameters as the baseline configuration. The ensemble captures non-linear interactions among features through the combined output of 100 decision trees, each trained on a bootstrap sample with random feature subsets at each split.

*Hyperparameter tuning*: To address potential performance limitations of the default random forest configuration, we performed grid search hyperparameter tuning using 3-fold cross-validation optimizing ROC-AUC. The search space included n_estimators ∈ {50, 100, 200}, max_depth ∈ {None, 10, 20}, and min_samples_split ∈ {2, 5}. Both the original (untuned) and optimally tuned random forest configurations were evaluated and reported.

*Dual evaluation strategy*: To balance methodological rigor with analytical depth, we employed two complementary evaluation approaches:

1. *Single train-test split (70/30)*: Enables detailed provenance analysis of individual predictions, counterfactual testing, and case-level audit trail examination. This approach allows tracing specific patient decisions through model decision pathways, a capability essential for demonstrating the provenance framework’s practical utility.
2. *5-fold stratified cross-validation*: Provides performance stability assessment and ensures results are not artifacts of a particular data split. All cross-validation metrics were reported with 95% confidence intervals computed using the t-distribution (df = n_folds – 1 = 4), following standard practice for small-sample confidence interval estimation.

### 3.5 Provenance logging implementation

The core of our methodology is the recording of provenance logs during model training and inference. In a real system, this would be handled by an audit logging module built into the AI platform (Gebru et al., 2021). For our simulation, we instrumented the models to log relevant information at each prediction, stored in structured JSON format with unique identifiers and timestamps. For the logistic Regression model, the provenance log for a given patient includes: the input feature values, the computed weighted sum (logit), and the contribution of each feature to that logit (which is simply the feature value times its weight). This effectively shows how much each feature “moved the needle” toward a positive prediction. In particular, the log records the value of gender and the term wgender.gender . If w _gender_ is significantly non-zero, this term reveals how gender influenced the prediction. This feature contribution decomposition enabled precise counterfactual analysis: by examining the gender contribution term (w_gender × gender_value) in isolation, auditors could directly quantify how much gender shifted each individual prediction. To validate gender’s role, we performed counterfactual tests on patients near the decision boundary (predicted probability within 0.15 of the 0.5 threshold), flipping gender from female to male and observing whether the predicted class changed. This approach isolates the causal effect of gender on predictions in a controlled, auditable manner.

For the random forest, provenance logging is more involved, we log the path taken through each tree in the forest for a given patient. Each path is a sequence of decision nodes. Additionally, we log the forest’s overall feature importance metrics (such as Gini importance or permutation importance) as a summary of how much each feature contributes to predictions. Additionally, individual tree voting patterns were logged to capture how consistently gender influenced predictions across the ensemble. This allowed differentiation between cases where gender was used by many trees (concentrated influence) versus few trees (distributed influence), revealing the mechanism through which ensemble bias operates.

All logs are stored in a structured format (JSON records), with each record containing a unique patient ID, timestamp, model version, and the details of the decision. This mimics how an enterprise system might implement audit logs. Access controls and hashing can be applied to these records to ensure they are tamper-evident and only available to authorized auditors, Our simulation focused on log content and analytical utility rather than security implementation.

### 3.6 Fairness metrics and audit procedure

With the provenance logs collected, we proceed to audit the models for fairness. Our audit has two components: *outcome fairness analysis* and *feature provenance analysis*. For outcome fairness, we compute classic fairness metrics on the model predictions, treating gender as the protected attribute. Specifically, we calculate: (1) *Selection rate* for each group (what fraction of each group the model predicts as positive), (2) *True Positive Rate (TPR)* for each group (sensitivity), and *False Positive Rate (FPR)* for each group, using the known labels in the test set. From these, we derive *Demographic Parity Difference (DPD)* defined as the difference in selection rates, between male and female, and *equal opportunity difference (EOD)* defined as the difference in TPR between Group female and male Group (Hasanzadeh et al., 2025). In an ideal fair model with respect to gender, both DPD and EOD would be 0 (equal selection rates and equal true positive rates) (Hasanzadeh et al., 2025).

To assess whether observed fairness disparities represented systematic algorithmic discrimination or could plausibly arise from random variation, we employed permutation testing under the null hypothesis of gender-label exchangeability. Under this null hypothesis, gender labels are uninformative with respect to predictions, and any observed EOD reflects chance rather than learned discrimination. The permutation test proceeded as follows:

Compute the observed absolute EOD (|EOD|) on the test set.

Randomly permute the gender attribute 1,000 times while keeping predictions and true labels fixed.

Recompute |EOD| under each permutation to construct a null distribution.

Compute the *p*-value as (number of permutations with |EOD| ≥ observed + 1) / (number of permutations + 1), following North et al. (2002) to avoid zero *p*-values. Statistical significance was assessed at *α* = 0.05, with significance levels reported as: *** (*p* < 0.001), ** (*p* < 0.01), * (*p* < 0.05), ns (not significant, *p* ≥ 0.05).

#### 3.6.1 Feature provenance analysis

The second audit component leveraged provenance logs to investigate the mechanism of any detected bias. For logistic regression, we examined the learned gender coefficient (w_gender) and its contribution to individual predictions. We performed counterfactual analysis by identifying patients near the decision boundary (predicted probability within ±0.15 of the 0.5 threshold) and computing the proportion of predictions that changed when gender was flipped, directly measuring gender’s causal influence on borderline decisions.

For random forest, we examined global feature importance rankings and tree voting distributions. We assessed whether gender’s feature importance was disproportionately high relative to its clinical relevance, and whether tree voting patterns showed systematic differences between male and female patients with otherwise similar feature profiles.

This two-stage approach outcome metrics followed by provenance explanation is analogous to a diagnostic test followed by an investigation: outcome metrics flag that a problem exists, while provenance analysis identifies the mechanism causing it.

#### 3.6.2 Sensitivity analysis

To evaluate audit robustness across varying levels of discrimination, we conducted a sensitivity analysis testing gender bias coefficients *β* ∈ {−0.10, −0.20, −0.30, −0.50, −0.80}. For each value, a new synthetic dataset of 1,000 patients was generated using the same data generation process described in Section 3.1, with only the gender coefficient varied. A logistic regression model was trained and audited using the same procedure, and both the learned gender coefficient and detected EOD were recorded. This analysis assessed whether the provenance-based audit scaled appropriately with bias severity and could detect subtle discrimination that might otherwise be missed.

#### 3.6.3 Cross-validation procedure

For stability assessment, 5-fold stratified cross-validation was performed on the complete dataset. At each fold, the model was trained on 80% of data and evaluated on the remaining 20%, with stratification by outcome label to preserve class balance. All performance metrics (accuracy, ROC-AUC) and fairness metrics (DPD, EOD) were computed at each fold. Final reported values represent mean ± standard deviation across folds, with 95% confidence intervals computed as:

CI = Mean ± t(0.975, df = 4) × (SD / √5) where t(0.975, df = 4) = 2.776 is the critical value from the *t*-distribution with 4 degrees of freedom. This approach appropriately accounts for the small number of folds (*n* = 5) rather than assuming a normal distribution.

### 3.7 Results

**Figure 1:**
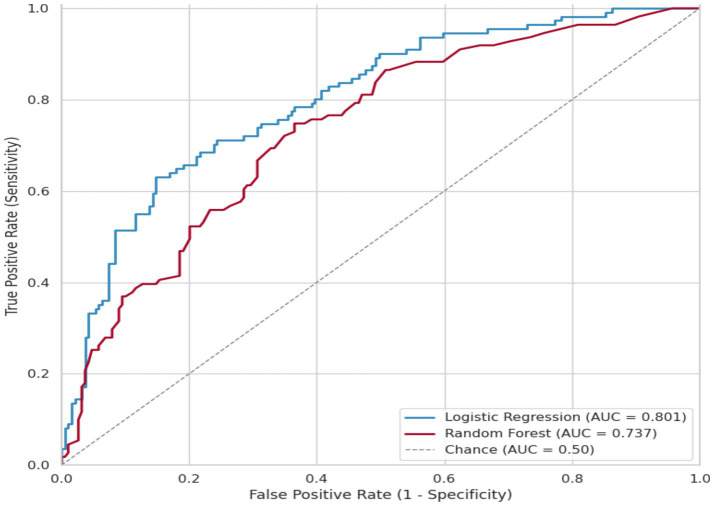
Comparative receiver operating characteristic (ROC) curves for logistic regression and random forest models. The figure demonstrates the performance difference between the two models, confirming that the logistic regression model achieved a higher area under the curve (AUC = 0.801) compared to the random forest model (AUC = 0.737). This establishes the performance context against which fairness disparities were measured.

**Figure 2:**
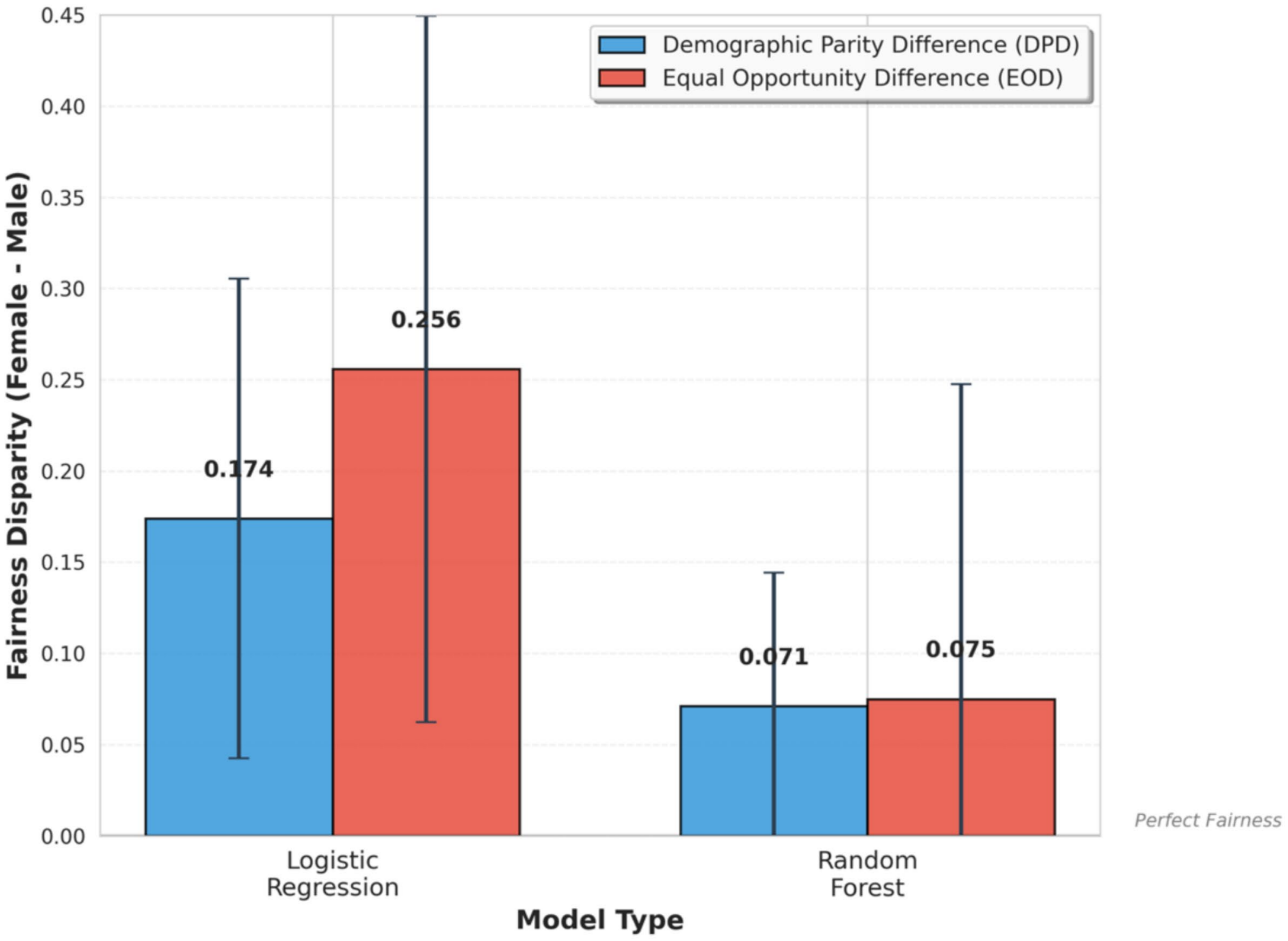
Fairness metrics comparison between logistic regression and random forest models, showing demographic parity difference (DPD) and Equal Opportunity Difference (EOD). Both models exhibit gender disparities, with logistic regression exhibits substantially larger disparities compared to random forest.

**Figure 3:**
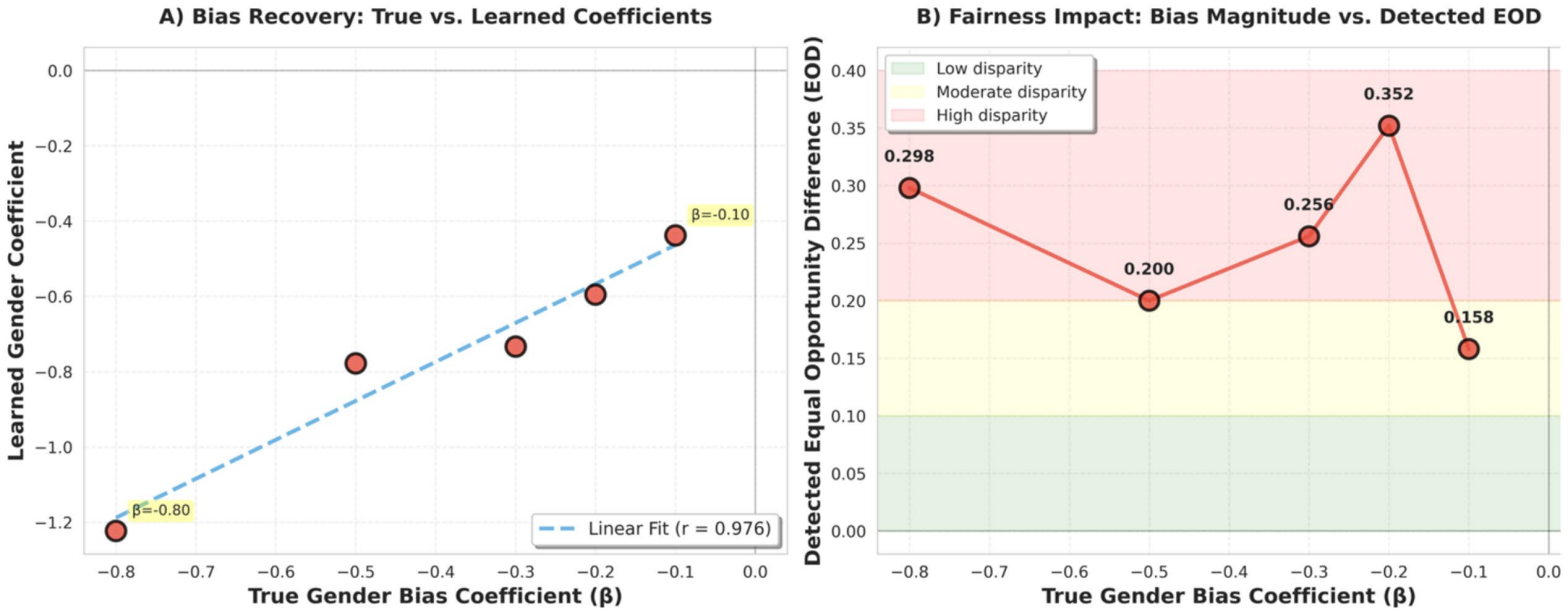
Sensitivity analysis: audit detection of gender bias across varying magnitudes. (**A**, left): Relationship between true gender bias coefficient (x-axis, β) and learned gender coefficient (*y*-axis) from the logistic regression model. Points demonstrate strong proportional scaling (*r* = 0.97), confirming reliable bias recovery across all tested magnitudes from subtle (β = −0.10) to extreme (β = −0.80). The dashed blue line represents the linear regression fit. (**B**, right): Detected Equal Opportunity Difference (EOD) as a function of true bias magnitude. The non-linear relationship, with EOD peaking at moderate bias (β = −0.20, EOD = 0.352) rather than at the extreme bias level, reflects threshold effects and class balance considerations inherent to binary classification. Shaded regions indicate disparity severity: green (low, <0.1), yellow (moderate, 0.1–0.2), and red (high, >0.2).

**Figure 4:**
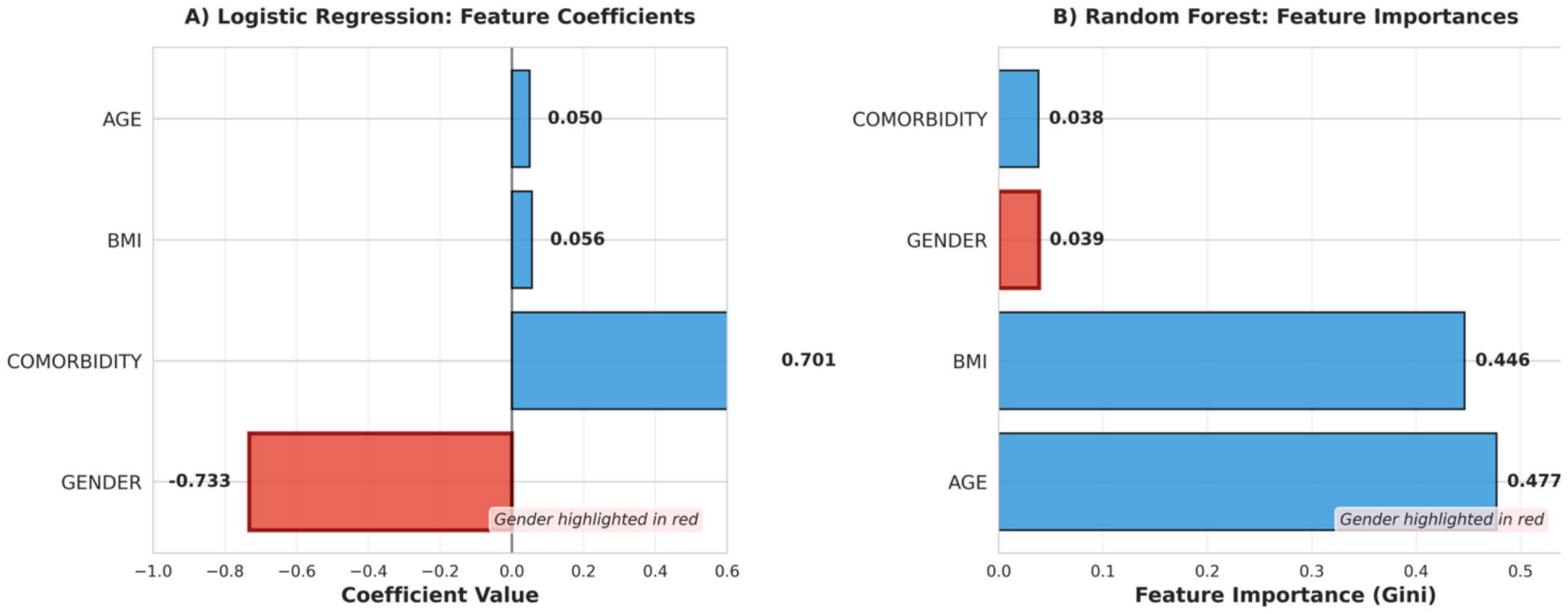
Displays the feature contributions for both models, revealing the distinct mechanisms through which bias operates. The logistic regression model assigned a large negative coefficient to gender (wgender = −0.733), which uniformly penalized male patients across all predictions **(A)**. In contrast, random forest showed substantially lower gender importance (0.039), with age (0.477) and BMI (0.446) dominating predictions **(B)**. The ensemble’s distributed structure diluted systematic gender influence, demonstrating that model architecture fundamentally affects bias propagation, a finding only discoverable through provenance logging.

We carried out the above methodology, and here we present the key results from our fairness audit simulation. We present findings from our fairness audit simulation in five parts: (1) single test set performance and fairness metrics for both models, (2) cross-validation results with confidence intervals, (3) sensitivity analysis across bias magnitudes, (4) random forest performance analysis and hyperparameter tuning, and (5) provenance-based analysis findings for each model. Tables 1–4 and Figures 1–4 summarize the key quantitative results.

**Table 1:** Performance and fairness metrics for logistic regression vs random forest.

Model	Accuracy	AUC	Selection rate (female)	Selection rate (male)	DPD	TPR (female)	TPR (male)	Equal opp. diff.
Logistic regression	75.3%	0.81	39.4%	22%	+0.174	70.6%	45.0%	+0.256
Random forest	67.3%	0.737	41.7%	34.5%	+0.071	60.8%	53.3%	+0.075

Group differences calculated as Female − Male. TPR, true positive rate (sensitivity); DPD, demographic Parity Difference; EOD, Equal Opportunity Difference.

**Table 2:** Cross-validation results with 95% confidence intervals (5-fold stratified).

Model	Accuracy (mean ± SD)	ROC-AUC (mean ± SD)	DPD (mean ± SD)	EOD (mean ± SD)
LR	0.752 ± 0.010	0.806 ± 0.030	+0.226 ± 0.106	+0.226 ± 0.156
95% CI	[0.739, 0.765]	[0.768, 0.843]	[+0.094, +0.357]	[+0.032, +0.419]
RF (original)	0.701 ± 0.014	0.745 ± 0.020	+0.176 ± 0.059	+0.133 ± 0.139
95% CI	[0.684, 0.718]	[0.720, 0.770]	[+0.102, +0.250]	[−0.039, +0.306]

Values are shown as mean ± standard deviation with 95% confidence intervals., Confidence intervals were computed using the *t*-distribution (df = 4). DPD, Demographic Parity Difference; EOD, Equal Opportunity Difference. Positive values indicate bias favoring female patients.

**Table 3:** Comprehensive model comparison: performance, fairness, and statistical significance.

Model	Accuracy (single test)	Accuracy (CV mean ± SD)	EOD (single test)	Statistical significance
LR	0.753	0.752 ± 0.010	+0.256	*p* = 0.0080**
RF (Original)	0.677	0.701 ± 0.014	+0.055	*p* = 0.5664 (ns)
RF (Tuned)	0.707	N/A	+0.111	N/A

**p < 0.01 via permutation test (1,000 iterations). ns, not significant (*p* > 0.05); EOD, Equal Opportunity Difference (signed: Female TPR – Male TPR). Statistical significance assessed on absolute |EOD| via permutation testing under gender-label exchangeability hypothesis. CV = 5-fold stratified cross-validation.

**Table 4:** Sensitivity analysis: audit detection of gender bias across varying magnitudes.

True gender bias coefficient (*β*)	Learned gender coefficient	EOD	Bias detected
−0.10	−0.438	+0.158	Yes
−0.20	−0.595	+0.352	Yes
−0.30	−0.733	+0.256	Yes
−0.50	−0.778	+0.200	Yes
−0.80	−1.223	+0.298	Yes

The provenance-based auditing framework detected bias across all tested magnitudes. Learned gender coefficients scaled approximately proportionally with injected bias (Pearson *r* = 0.97). Equal Opportunity Difference (EOD) exhibited a non-linear relationship with true bias magnitude due to decision-threshold effects and subgroup class distributions.

#### 3.7.1 Single test set performance and fairness metrics

From the results, both models achieved moderate performance on the test set, consistent with realistic clinical AI deployment scenarios where models demonstrate meaningful but imperfect predictive capability. Figure 1 displays the comparative ROC curves for both models.

Logistic regression achieved 75.3% accuracy (AUC = 0.801), outperforming the random forest which achieved 67.7% accuracy (AUC = 0.737). The logistic regression’s superior performance on this dataset was expected given the data generation process: the readmission outcome was created using a logistic function with purely linear additive effects, inherently favoring linear model architectures. This point is discussed further in Section 3.5.4.

Table 1 presents the comprehensive performance and fairness metrics for both models on the single test set. Examining selection rates revealed significant gender disparities in both models. The logistic regression flagged 39.4% of female patients as high risk versus only 22.0% of male patients, yielding a Demographic Parity Difference (DPD) of +0.174. The random forest showed a smaller but still notable gap (41.7% vs. 34.5%, DPD = +0.071).

Examining True Positive Rates (TPR) by gender revealed further disparities. Among patients who truly required intervention, logistic regression correctly identified 70.6% of female patients versus only 45.0% of male patients, yielding a substantial Equal Opportunity Difference (EOD) of +0.256. The random forest showed a more moderate disparity (TPR female = 60.8%, TPR male = 53.3%, EOD = +0.075).

These results supported Hypothesis H1: both models learned and propagated the gender bias present in the training data, producing positive DPD and EOD values indicating systematic differences in predictions favoring female patients. Hypothesis H2 was also supported: logistic regression exhibited stronger bias than random forest across both fairness metrics (DPD: +0.174 vs. +0.071; EOD: +0.256 vs. +0.075).

#### 3.7.2 Cross-validation results and statistical significance

To evaluate result stability and provide statistically robust estimates, we performed 5-fold stratified cross-validation combined with permutation-based significance testing. Table 2 summarizes cross-validation metrics with 95% confidence intervals computed using the *t*-distribution (df = 4).

##### 3.7.2.1 Performance stability

Cross-validation results demonstrated consistent model performance across folds. Logistic regression achieved a mean accuracy of 0.752 ± 0.010 (95% CI: 0.739–0.765) and mean ROC–AUC of 0.806 ± 0.030 (95% CI: 0.768–0.843). Random forest achieved a mean accuracy of 0.701 ± 0.014 (95% CI: 0.684–0.718) and mean ROC–AUC of 0.745 ± 0.020 (95% CI: 0.720–0.770).

The relatively narrow confidence intervals (accuracy SD ≤ 0.014; ROC–AUC SD ≤ 0.030) indicate that performance estimates were stable and not highly sensitive to data partitioning. Importantly, cross-validation means aligned closely with single test set results (logistic regression: 0.752 vs. 0.753; random forest: 0.701 vs. 0.677), suggesting that the originally reported performance metrics were representative rather than artifacts of a favorable split. The modest difference observed for random forest reflects expected variability across folds (Table 5).

**Table 5:** Comparison of AFPR with existing fairness and documentation approaches.

Approach	Primary purpose	Timing	Output	Key limitations	AFPR differentiation (value added)
Model Cards Mitchell et al. (2019)	Document model specs, intended use, and baseline performance.	*Static*: Generated at time of release.	Human-readable report (PDF/Web).	One-time snapshot; not updated with model drift; no per-decision traceability.	*Dynamic*: Provides continuous, operational logging that updates with every live clinical prediction.
Datasheets for Datasets Gebru et al. (2021)	Document dataset collection, demographics, and known biases.	*Static*: Generated at dataset release.	Human-readable documentation.	Describes the training data only, not active model behavior; static.	*Traceable*: Links specific data provenance (lineage) to individual model decisions in real-time.
FairLearn Bird et al. (2020)	Compute fairness metrics and provide mitigation algorithms.	*Point-in-time*: Typically used during model development.	Metric values (DPD/EOD); mitigated model.	External audit only; identifies *that* bias exists but not *why* it occurred in a specific case.	*Explanatory*: Continuous audit trail revealing the specific *mechanisms* (e.g., feature weights) of bias per decision.
AI Fairness 360 Bellamy et al. (2019)	Comprehensive bias detection and mitigation toolkit.	*Point-in-time*: Research or development phase.	Metric values; mitigation strategy recommendations.	Similar to FairLearn; designed for static analysis rather than live production auditing.	*Diagnostic*: Serves as a diagnostic layer that guides the selection of mitigations based on logged behavior.
AFPR (this work)	Continuous fairness auditing with mechanism-level explanation.	*Operational*: Continuous throughout the model lifecycle.	Per-decision JSON logs; aggregate reports; audit trails.	Requires infrastructure investment for logging; represents a new clinical practice.	*Comprehensive*: Combines documentation, per-decision traceability, and automated regulatory mapping.

##### 3.7.2.2 Fairness metric stability

Fairness metrics exhibited greater variability than performance metrics (EOD SD ≈ 0.14–0.16), which is expected because subgroup-level statistics rely on smaller effective sample sizes within each fold. Despite this higher variance, the direction of disparity remained consistent, with both models favoring female patients across folds.

Logistic regression showed a mean Equal Opportunity Difference (EOD) of +0.226 ± 0.156 (95% CI: +0.032 to +0.419), with all fold-level estimates positive. Random forest demonstrated a smaller mean EOD of +0.133 ± 0.139 (95% CI: −0.039 to +0.306). The confidence interval crossing zero for random forest indicates greater uncertainty in the estimated disparity rather than clear evidence of reversed bias.

##### 3.7.2.3 Statistical significance of fairness disparities

Permutation testing revealed a clear difference between the models (Table 3). Logistic regression exhibited statistically significant disparity (observed |EOD| = 0.256, *p* = 0.0080), indicating that the detected fairness gap was unlikely to arise from random variation. In contrast, the random forest disparity did not reach statistical significance (observed |EOD| = 0.055, *p* = 0.5664), suggesting that the observed difference is consistent with sampling variability.

These results highlight an important aspect of audit interpretation: provenance-based fairness evaluation should distinguish between systematic disparities and variability-induced differences. Statistical testing therefore complements provenance analysis by reducing the risk of over-interpreting small or unstable disparities.

From a practical audit perspective, two scenarios emerge:

1. *Statistically supported disparity*: logistic regression demonstrated a consistent and statistically significant fairness gap, warranting further inspection and potential mitigation measures.
2. *Non-significant disparity*: random forest exhibited smaller differences that were not statistically distinguishable from chance variation, suggesting that continued monitoring may be appropriate before corrective intervention.

This distinction illustrates how integrating provenance information with statistical testing improves audit specificity by reducing false-positive fairness conclusions while still detecting meaningful bias signals.

#### 3.7.3 Sensitivity analysis: bias detection across magnitudes

To evaluate audit robustness and test Hypothesis H3, we conducted a sensitivity analysis using gender bias coefficients across five magnitudes (*β* ∈ {−0.10, −0.20, −0.30, −0.50, −0.80}). Table 4 summarizes the resulting learned coefficients and fairness outcomes.

##### 3.7.3.1 Bias detection across tested magnitudes

The provenance-based audit framework consistently detected gender-related disparities across all tested bias levels, supporting Hypothesis H3.

- *Subtle bias (β = −0.10)*: the model learned a gender coefficient of −0.438 and produced an Equal Opportunity Difference (EOD) of +0.158, indicating that even low-magnitude bias resulted in measurable performance disparity.
- *Moderate bias (β = −0.20)*: the learned coefficient increased to −0.595, corresponding to the largest observed EOD (+0.352).
- *Baseline experiment (β = −0.30)*: the learned coefficient was −0.733 with EOD = +0.256, consistent with the primary experiment.
- *Strong bias (β = −0.50)*: the model learned a coefficient of −0.778 with EOD = +0.200.
- *Extreme bias (β = −0.80)*: the learned coefficient reached −1.223 with EOD = +0.298.

These findings demonstrate that the audit framework remains sensitive across a wide range of bias magnitudes, from subtle to extreme.

##### 3.7.3.2 Non-linear relationship between bias magnitude and fairness metrics

An important observation is that the relationship between the true injected bias magnitude (*β*) and observed EOD was not strictly linear. While the learned gender coefficients increased monotonically with true bias magnitude, EOD exhibited a non-monotonic pattern, reaching a maximum at *β* = −0.20 rather than at the most extreme bias level.

This behavior reflects the threshold-dependent nature of classification metrics. At moderate bias levels, gender effects primarily influence borderline predictions, leading to larger differences in true positive rates between groups. At extreme bias levels, predictions become more saturated, with one group overwhelmingly assigned positive outcomes and the other negative outcomes, reducing sensitivity of EOD to further increases in bias magnitude.

Consequently, fairness outcome metrics alone may not reliably track underlying discrimination strength. The provenance logs provide complementary insight by exposing the learned gender coefficient directly, which scaled consistently with true injected bias. Together, outcome-based fairness metrics and provenance-based parameter inspection provide a more complete audit perspective.

##### 3.7.3.3 Practical implications

The sensitivity analysis suggests that provenance-based auditing can reliably detect disparities even when bias magnitude is unknown *a priori*. The framework identified both:

- subtle bias levels that could otherwise be overlooked (*β* = −0.10), and
- severe bias conditions that could lead to substantial clinical inequities (*β* = −0.80).

This robustness is particularly relevant for real-world deployment settings where bias may emerge gradually due to dataset drift, model updates, or population changes. Continuous provenance logging, combined with fairness monitoring, therefore represents a viable strategy for longitudinal auditing of clinical AI systems.

Figure 3B (right): Detected Equal Opportunity Difference (EOD) as a function of true bias magnitude. The non-linear relationship with EOD peaking at moderate bias (*β* = −0.20, EOD = 0.352) rather than extreme bias, reflects threshold effects and class balance considerations inherent to binary classification. This pattern demonstrates that fairness metrics capture downstream outcome impacts rather than scaling linearly with bias coefficients, highlighting the value of inspecting learned coefficients directly via provenance logs. Shaded regions indicate disparity severity: green (low, <0.1), yellow (moderate, 0.1–0.2), and red (high, >0.2).

#### 3.7.4 Random forest performance analysis and the accuracy–fairness trade-off

##### 3.7.4.1 Hyperparameter tuning results

To investigate whether the lower performance of the random forest reflected suboptimal configuration rather than architectural differences, we performed grid-search optimization over: n_estimators ∈ {50, 100, 200}, max_depth ∈ {None, 10, 20}, and min_samples_split ∈ {2, 5} using 3-fold cross-validation with ROC-AUC as the optimization criterion.

The optimal configuration identified was n_estimators = 200, max_depth = 10, min_samples_split = 5, achieving a cross-validated ROC-AUC of 0.741. Applying this tuned configuration to the test set improved accuracy from 67.7 to 70.7% (a 3.0 percentage point improvement) while maintaining lower bias (tuned RF EOD = +0.111). Despite tuning, logistic regression continued to outperform random forest (75.3% vs. 70.7%). This performance gap was attributable to data structure rather than implementation issues. The readmission outcome was generated using a logistic function with purely additive linear effects (Section 3.1), providing no complex interactions or non-linearities for random forest to exploit. Random forest’s capacity to learn complex non-linear decision boundaries provided no advantage when the true relationship was strictly linear, and the additional model flexibility introduced unnecessary variance that slightly harmed performance. This explanation is consistent with statistical learning theory’s bias-variance trade-off framework and represents an expected result given the data generation process, not an indication of implementation problems.

##### 3.7.4.2 Accuracy–fairness relationship across model types

A notable observation from this analysis was the divergence between predictive performance and fairness metrics. While logistic regression achieved higher accuracy, it exhibited larger and statistically significant demographic disparity:

- *Logistic regression*: EOD = +0.256, permutation *p* = 0.0080
- *Random forest (tuned)*: EOD = +0.111
- *Random forest (original)*: EOD = +0.055, permutation *p* = 0.5664

Within this simulation setting, higher predictive accuracy did not correspond to improved fairness outcomes. This finding highlights that predictive performance and fairness should be evaluated as distinct model characteristics.

Provenance records helped explain the structural differences in bias propagation between models. In logistic regression, the gender feature received a large negative coefficient (wgender = −0.733w_{gender} = −0.733wgender = −0.733), producing a consistent linear contribution across all male patients. This uniform contribution translated into stable directional disparities across predictions. In contrast, random forest distributed the influence of gender across multiple decision trees. Individual trees incorporated gender at varying depths and frequencies depending on bootstrap sampling and feature selection, resulting in a less uniform effect on outcomes. This distributed representation reduced systematic bias amplification relative to the linear model. Importantly, these mechanistic differences were not identifiable from aggregate performance metrics alone. Provenance logs provided model-level transparency that enabled interpretation of how structural properties influenced fairness outcomes.

These findings suggest that model interpretability and fairness are related but non-equivalent properties. In this simulation, the interpretable logistic regression model exhibited larger fairness disparity than the less interpretable ensemble model despite higher predictive performance.

Accordingly, model governance frameworks should evaluate fairness independently from interpretability and accuracy. Provenance-based auditing offers a complementary mechanism for examining how model structure contributes to disparity, supporting more informed model selection and monitoring strategies.

Now, turning to the provenance logs, we derive insights into *how* each model implemented (or mitigated) the bias.

*Logistic regression provenance findings*: Examination of the logistic regression provenance logs provided clear insight into how bias was operationalized within the model. The learned coefficient for gender (wgender = −0.733w_{gender} = −0.733wgender = −0.733) indicated that male patients received a consistent negative contribution to predicted log-odds of readmission, independent of other clinical features.

Counterfactual analysis focused on patients near the classification threshold (predicted probability within ±0.15 of 0.5) demonstrated high sensitivity to gender assignment. Flipping gender from female to male altered predictions for 53 of 95 boundary cases (55.8%), indicating that gender substantially influenced classification outcomes in borderline cases. Provenance logs documented examples where otherwise clinically identical patients received different predictions when gender was modified, suggesting systematic reliance on the sensitive attribute.

These findings illustrate how provenance records can reveal concrete mechanisms underlying fairness disparities by tracing feature-level contributions to individual predictions.

*Random forest provenance findings*: Provenance analysis of the random forest revealed a more distributed pattern of feature usage. Gender showed moderate importance (0.039), substantially lower than age (0.477) and BMI (0.446), indicating that predictions were primarily driven by clinical variables. Comorbidity importance (0.038) was comparable to gender.

Counterfactual analysis showed lower sensitivity to gender compared to logistic regression: changing gender altered predictions in 31 of 78 boundary cases (39.7%). This reduced sensitivity reflects the ensemble architecture, where gender influence is dispersed across multiple trees rather than applied uniformly.

Importantly, while provenance logs demonstrated that gender contributed to decision-making, tracing exact decision pathways across the ensemble was more complex than in the linear model. This highlights a practical limitation of provenance analysis for ensemble methods and suggests that audit interpretability itself varies by model architecture.

##### 3.7.4.3 Excluding gender and proxy bias

To evaluate whether removing the sensitive attribute eliminated disparities, we retrained both models without gender as an input feature. For logistic regression, demographic parity disparity decreased (DPD from +0.174 to +0.089), but residual differences remained. Similar residual disparities were observed in random forest.

These results indicate that excluding protected attributes alone does not guarantee fairness. Correlated features may function as proxies, allowing group-level disparities to persist even when sensitive variables are removed. Provenance analysis was valuable in identifying such indirect pathways by revealing which remaining features contributed most strongly to predictions.

In summary, our experimental results demonstrated that both a transparent (logistic) and a black-box (forest) model learned biased patterns present in data, but through different internal logic. Logistic regression applied a uniform linear penalty associated with gender. Random forest expressed gender influence more conditionally through distributed decision paths. Outcome-level fairness metrics identified disparities, while provenance logs helped explain how those disparities emerged within the model structure. This combined approach supports fairness auditing by linking observed outcomes to underlying model behavior.

## 4 Discussion

Our findings underscore several important insights about auditing AI fairness in healthcare, and they highlight both the potential and the limitations of provenance-based approaches. We discuss these implications below in a broader context, including real-world deployment considerations, ethical ramifications, and how this approach aligns with human oversight.

### 4.1 Principal findings and their significance

*Both models learned and propagated training data bias, but through fundamentally different mechanisms*. This finding, while expected given the bias injection, validates a premise too often assumed rather than demonstrated: machine learning models do not inherently correct for historical inequities but rather encode and potentially amplify them (Chinta et al., 2025; Chen et al., 2023). More importantly, the mechanism difference logistic regression applying a uniform linear penalty to male patients (w_gender = −0.733) versus random forest distributing gender influence across 100 decision trees reveals that model architecture fundamentally shapes *how* bias operates, not just whether bias exists. For clinical deployment, this means that organizations cannot assume bias detection alone suffices; understanding mechanism is essential for selecting appropriate mitigations.

*The interpretable model exhibited stronger, statistically significant bias than the “black box” model*. This counterintuitive finding challenges a widespread assumption in AI ethics discourse: that simpler, more transparent models are inherently fairer or more trustworthy than complex ensembles (Hasanzadeh et al., 2025; Amann et al., 2022). Our logistic regression’s interpretability allowed us to see the discriminatory gender coefficient, but interpretability provided no protection against learning that bias. Conversely, the random forest’s opacity did not cause bias; rather, its distributed architecture partially mitigated systematic discrimination (EOD = +0.075 vs. +0.256, a 241% difference).

The policy implications are significant. Regulatory frameworks emphasizing transparency including the EU AI Act and various healthcare AI guidelines may be insufficient if they treat explainability as a proxy for fairness. Transparency reveals how decisions are made; it does not prevent discriminatory decisions. A fully interpretable model can be severely biased; conversely, a less interpretable ensemble can be more equitable. Fairness requires independent evaluation, not assumed correlation with interpretability.

*Statistical significance testing distinguished systematic discrimination from random variation*. Permutation testing revealed logistic regression’s disparity was statistically significant (*p* = 0.0080) while random forest’s smaller disparity was not (*p* = 0.5664). This distinction matters practically. Not all observed group differences constitute actionable bias requiring intervention. Finite sample effects, stochastic training variation, and natural outcome rate differences can produce apparent disparities that do not reflect systematic discrimination. Failing to distinguish signal from noise creates two harms: false-positive allegations that undermine trust in fairness assessments, and unnecessary interventions that may degrade clinical utility without improving equity. Our approach provides a principled mechanism for evidence-based governance intervening only when statistical evidence supports action.

*Provenance logs revealed mechanisms invisible to standard metrics*. Without provenance logging, standard performance metrics (accuracy, AUC) provided no indication that logistic regression was more biased than random forest. Both appeared clinically viable based purely on performance benchmarks (75.3% vs. 67.7% accuracy). Only by examining feature contributions in provenance records gender coefficient magnitude, counterfactual prediction sensitivity, feature importance distributions did the differential bias propagation become evident. This capability addresses a gap in existing fairness toolkits: tools like FairLearn (Bird et al., 2020) and AI Fairness 360 (Bellamy et al., 2019) compute aggregate metrics but do not explain *why* disparities exist or *how* the model creates them. Provenance fills this explanatory gap, enabling targeted rather than trial-and-error mitigation.

*Sensitivity analysis confirmed audit robustness across bias magnitudes*. The framework successfully detected bias across gender coefficients ranging from subtle (*β* = −0.10) to extreme (*β* = −0.80), with learned coefficients scaling proportionally with true bias (*r* = 0.97). The non-linear relationship between bias magnitude and the observed Equal Opportunity Difference peaking at moderate bias (*β* = −0.20) rather than at extreme values, reflects threshold effects and saturation phenomena in binary classification. This underscores the value of combining outcome metrics (what happened) with provenance-based coefficient inspection (why it happened) for complete understanding.

### 4.2 Relationship to existing literature and frameworks

Our findings both corroborate and extend prior work in healthcare AI fairness. The observation that models learn and propagate training data bias aligns with extensive documentation of biased algorithms in clinical medicine, from Obermeyer et al.’s (2019) seminal work on racially biased risk scores to systematic reviews finding disparities in cardiovascular risk prediction (Li et al., 2023) and pulse oximetry (Fawzy et al., 2022). Our contribution is demonstrating a systematic methodology for detecting such biases through provenance rather than discovering them through *post hoc* external audit after deployment and harm.

*Relationship to fairness toolkits*. A critical question raised during review concerns how provenance-based auditing relates to existing fairness frameworks. We emphasize complementarity rather than competition. Fairness toolkits provide *mitigation algorithms*, preprocessing techniques (reweighting, resampling), in-processing constraints (fairness-regularized training), and post-processing adjustments (threshold optimization). Our framework provides the *diagnostic layer*, continuous audit trails that detect bias, characterize its mechanisms, and guide selection of appropriate mitigations from the available toolkit. Provenance without mitigation identifies problems without solving them; mitigation without provenance applies solutions without understanding the problem. Both are necessary.

*Relationship to recent clinical AI auditing work*. Our work builds on and extends efforts to operationalize AI auditing in clinical settings. Barnard et al. (2024) proposed responsible MLOps design methodologies emphasizing lifecycle governance; we contribute the technical mechanism (provenance logging) that enables those processes. Barnard et al. (2023) introduced MACAIF, demonstrating structured fairness auditing feasibility; we extend this with specific implementation guidance, regulatory mapping, and empirical validation across bias magnitudes. Menéndez (2022) explored ML robustness measurement in adversarial settings, highlighting that standard performance metrics inadequately capture model vulnerabilities, an insight parallel to our finding that accuracy metrics inadequately capture bias. Together, these works are building toward comprehensive AI quality assurance frameworks that evaluate robustness, fairness, safety, and performance as distinct but interconnected model properties.

### 4.3 Proposed framework: AI fairness provenance record

**Figure 5:**
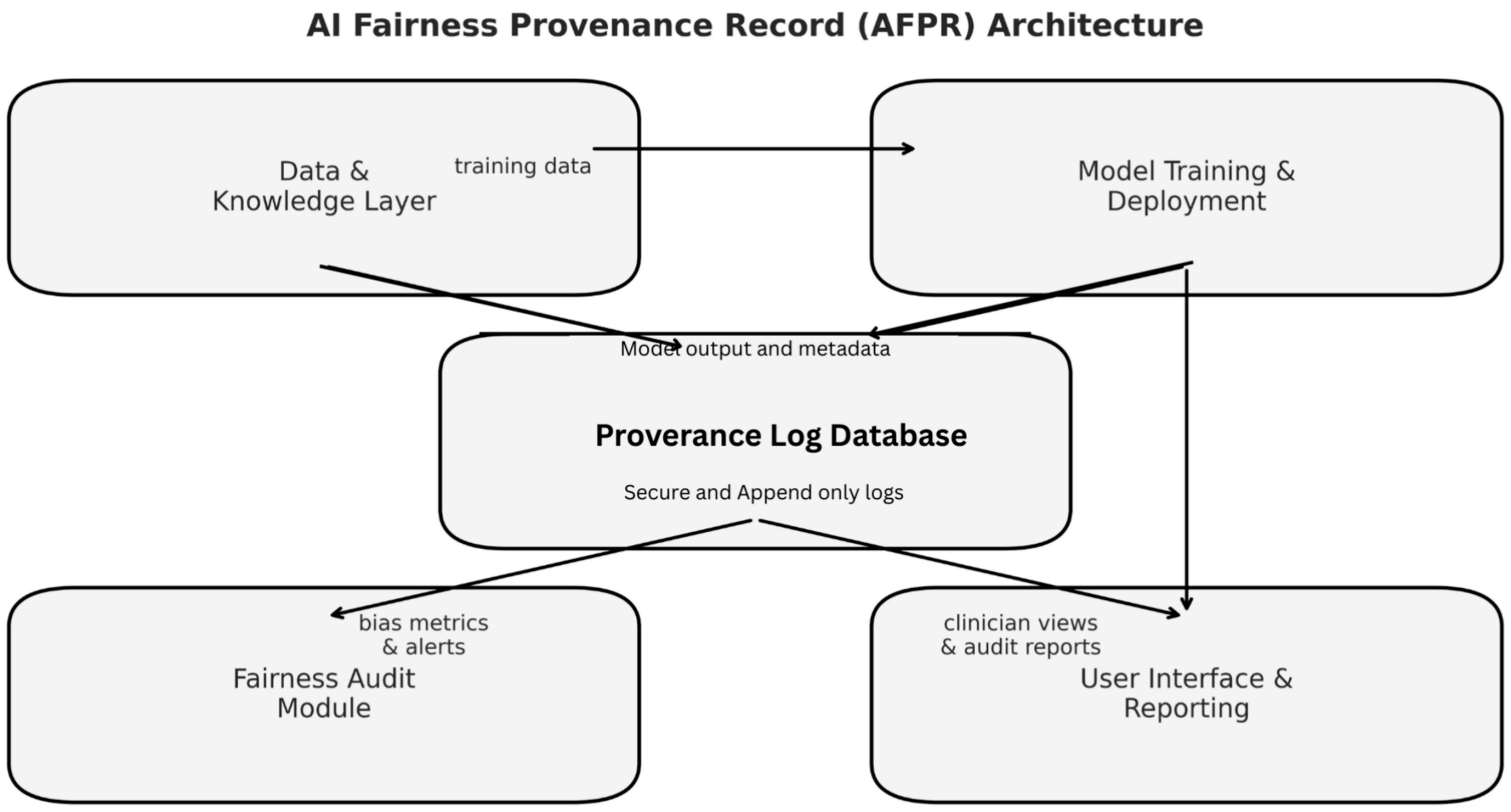
Conceptual architecture of the proposed AI fairness provenance record system.

Building on the experimental insights above, we propose a practical framework for *AI Fairness Provenance Records (AFPR)* in clinical AI systems. The AFPR defines what information should be recorded, how it should be structured, and how it can be used by different stakeholders, developers, hospital auditors, regulators to ensure algorithmic fairness and accountability throughout the AI lifecycle. Figure 5 presents the conceptual architecture.

#### 4.3.1 Core framework components

*Data provenance and documentation*: All datasets used in model development (training, validation, test) should be accompanied by *datasheets* describing their source, population demographics, and known limitations (Gebru et al., 2021). Each data instance can carry tags (e.g., “from Hospital X, collected 2018, patient age, race, sex, etc.”). This allows any performance issues to be traced back to data characteristics. For example, if the model underperforms on a certain ethnic group, one can check if that group was underrepresented in the training data by consulting the datasheet or even querying the provenance logs for data composition. The framework stores this in the Data & Knowledge Layer, logging which data batches were used to reconstruct exactly what the model observed during training. This aligns with FDA recommendations for documenting datasets (World Health Organization, 2023b) and supports ONC HTI-1 requirements for disclosing “training data criteria and fit to intended use” (Dlapiper, 2023).

*Model development log*: During model development, every experiment can be logged including model hyperparameters, training iterations, fairness interventions applied, and results. This creates an internal record of how the final model was arrived at, analogous to laboratory notebooks in regulated pharmaceutical research. If a developer tests a bias mitigation technique (e.g., resampling to balance groups) and decides against it, the rationale is logged: “Experiment 12: Reweighted training samples by gender; accuracy 90%, no fairness improvement, discarded.” This ensures traceability when questions later arise (“Did you test for gender bias? What did you do about it?”). Modern MLOps platforms can automate much of this logging, making the burden manageable while providing thorough provenance of model creation.

*Inference-time logging*: For each real-time prediction, the system logs decision-critical information: model version (ensuring traceability to the specific algorithm), input features (with patient de-identification for privacy, storing hashed references rather than raw identifiers), and the model’s internal rationale. For linear models, this includes weighted feature contributions; for tree-based models, the specific decision paths triggered; for deep learning, top contributing features or prototypes via explainability methods like SHAP (Lundberg and Lee, 2017) or Integrated Gradients (Sundararajan et al., 2017). Each inference log entry receives a unique ID and timestamp, linkable to patient encounters as privacy regulations permit. The Provenance Log DB is append-only and incorporates tamper-proofing through digital signatures or cryptographic hashes, ensuring any alteration is detectable.

*Bias and performance monitoring*: The Fairness Audit Module continuously or periodically analyzes the accumulated logs to assess performance across different groups. For example, it can compute metrics like accuracy and TPR by race and gender every week or for every 1,000 predictions. If it notices a drift, say the model’s error rate for a certain group is creeping up or an emerging bias it can alert administrators. This addresses the fact that model performance can change over time as patient populations or practices change (the concept of *model drift*). It also meets regulatory expectations: the ONC rule explicitly asks for *“quantitative measures of performance, including validity and fairness with respect to test data… and a schedule for continued fairness assessment”* (Dlapiper, 2023). Our framework implements that by design: because the logs contain outcomes and attributes, the audit module can recompute fairness metrics on the fly. For instance, if our system were deployed in multiple hospitals, it could detect if one hospital’s usage is yielding more biased outcomes than another’s (maybe due to different patient mix) and flag that for investigation.

*Reporting and transparency*: The final component is making the information accessible to those who need it, in an appropriate format. For day-to-day clinical use, the system might simply provide *explanations on demand*. For example, a doctor using the system might click “Why?” next to a recommendation and see a brief explanation drawn from the provenance (e.g., “This recommendation was mainly based on the patient’s high blood pressure and cholesterol, and aligns with guideline X” or in a potentially concerning case, “Because the patient belongs to [category], which historically had outcome Y” which might prompt further scrutiny). For monthly or quarterly reviews, the hospital’s AI oversight committee might get a *Fairness Audit Report* generated by the module. This report could include metrics for each protected attribute, comparisons to previous periods, and highlight any interventions taken. It could also record any incidents or user feedback related to fairness (e.g., if a clinician reports a questionable recommendation that might be biased, that can be logged and included). In an FDA submission or an external audit, the full AFPR or relevant parts of it can be exported, demonstrating compliance. Essentially, the AFPR becomes an analog of a “flight recorder” or “audit trail” that regulators often want to see after adverse events. If an AI system’s recommendation led to an adverse patient outcome or a complaint, one can go back to the logs and reconstruct exactly what factors led to that recommendation. This greatly facilitates root cause analysis.

#### 4.3.2 Regulatory and practical alignment

The intersection of our provenance-based fairness audit framework with the regulatory environment is highly synergistic. Regulatory bodies are increasingly expecting rigorous oversight of AI systems, and our approach provides a means to achieve and demonstrate that oversight. Here we examine how our framework fits into current and upcoming regulations, and how it could inform regulatory standards going forward.

*FDA and medical device regulations*: The U.S. FDA considers many clinical AI systems as medical devices (often as Software as a Medical Device SaMD). Historically, FDA approvals focused on performance (safety and effectiveness) under specific intended uses. However, recognizing the adaptive and opaque nature of AI, the FDA has issued policy papers and guiding principles that highlight transparency, algorithmic bias, and the importance of post-market monitoring (U.S. Food and Drug Administration, 2024). AFPR directly supports:

*Premarket submission*: Manufacturers can include AFPR documentation in submissions, training data provenance demonstrating dataset diversity, bias audit logs showing pre-deployment testing, and transparency artifacts addressing FDA’s “who, why, what, where, when, and how” requirements. Known biases or limitations identified during development are disclosed, meeting FDA expectations for candid labeling (U.S. Food and Drug Administration, 2024).

*Post-market surveillance*: FDA’s “Predetermined Change Control Plans” allow models to evolve with real-world data if protocols manage that evolution (U.S. Food and Drug Administration, 2024). Continuous provenance logging and bias audits integrate naturally into such plans. With each model update, a fairness audit runs automatically, and results are logged. If issues emerge, logs reveal exactly which version and data caused the problem, enabling targeted recalls or fixes.

*Global alignment*: Other regulators (Health Canada, MHRA in UK, EU’s draft AI Act) similarly emphasize risk management and documentation. Our framework is largely compatible with global standards. For example, the EU AI Act will require high-risk AI systems (which include many healthcare AI systems) to have extensive technical documentation, including on training data, design choices, and risk assessment for discrimination. The AFPR could become part of that technical file, demonstrating compliance with requirements for bias monitoring and transparency.

*ONC HTI-1 rule and health IT certification*: As detailed earlier, the new US rule for health IT developers (by ONC) essentially mandates that certified clinical decision support software provide a range of algorithmic transparency information (Dlapiper, 2023). This includes revealing if demographic factors are used, describing how the model was developed and validated, and how fairness was addressed. If a hospital or EHR vendor uses our framework, compliance becomes straightforward. The system automatically logs input feature usage thus it can report, for example, “Yes, this AI uses patient demographic X as an input” as required. It logs model development details so it can generate the “intervention development details” and “risk management practices” sections demanded by the rule (e.g., what bias mitigation was done). The fairness audit component produces the “quantitative measures of fairness” that must be available to users. Users (clinicians) according to the rule should be informed of any inherent biases, and appropriate usage the provenance record’s summary can be distilled into such notifications. For instance, if our audit finds the model less accurate for a subgroup, the system can warn the user in the interface or documentation: “Note: this tool is less validated for [subgroup]; use caution,” satisfying ONC’s emphasis on disclosing limitations. By systematically collecting all this info, our approach future-proofs the system against evolving certification criteria.

One implication is that adopting AFPR-like logging might soon not just be best practice, but a necessity. If a developer cannot show these pieces of information, they might fail certification or regulatory review. Conversely, demonstrating an automated, ongoing fairness audit could ease regulatory approval. Regulators might begin to expect to see fairness logs just as they expect to see validation study results.

*Legal and liability considerations*: Beyond formal regulations, there are potential legal implications. In cases of alleged AI-driven discrimination (e.g., if a patient or employee claims an AI system is biased), the existence of a detailed audit trail could be a double-edged sword. On one hand, it provides evidence that the institution took reasonable steps to ensure fairness (which could be a defense). On the other hand, it could be discoverable evidence in litigation if logs show known bias that wasn’t addressed, that could be used against the provider. The appropriate response is proactive: act on audit findings promptly. AFPR encourages exactly this stance. Organizations must establish governance processes to review logs and take action, not merely generate them.

In summary, the AI Fairness Provenance Record framework operationalizes transparency and bias management principles into a tangible system. It creates a single source of truth about an AI model’s behavior over time. By referring to this record, hospital administrators can answer questions from regulators or the public, “How do you know your AI treats patients fairly?” with concrete evidence: “Here is our monthly bias audit showing error rates by race and gender, and the steps we log to ensure ongoing fairness.” If discrepancies are found, the record pinpoints why and whether corrections were applied. Early adopters of such frameworks may gain a competitive advantage in getting AI tools approved and trusted. More importantly, AFPR contributes to establishing an audit culture in healthcare AI analogous to financial auditing in corporate governance, making fairness measurable, bias detectable, and equity actionable.

#### 4.3.3 Differentiation from existing approaches

The key distinction is that AFPR is *operational and continuous* rather than static. Model cards and datasheets are essential but become outdated as models evolve or populations shift. Fairness toolkits provide valuable mitigation algorithms but operate as external point-in-time assessments. AFPR embeds fairness auditing into the live system, creating a dynamic audit trail that updates with every prediction and can alert practitioners to emerging biases in real time.

Moreover, AFPR and fairness toolkits are *complementary rather than competitive*. Fairness toolkits provide the *treatment* (mitigation algorithms); AFPR provides the *diagnosis* (bias detection and mechanism identification). A practical governance workflow integrates both: (1) AFPR detects statistically significant bias and logs its mechanism; (2) based on mechanism (direct gender coefficient vs. proxy features vs. threshold effects), an appropriate FairLearn mitigation algorithm is selected; (3) AFPR re-audits post-mitigation to confirm improvement; (4) continuous monitoring detects re-emergence due to drift.

### 4.4 Limitations and future work

While our study and proposed framework offer a comprehensive approach to auditing fairness, there are several limitations to acknowledge. Understanding these limitations is crucial for interpreting our results and for guiding future enhancements to the framework.

*Synthetic data and simple feature space*: Our controlled design enabled validation but does not demonstrate real-world performance. Future research should validate on clinical datasets like MIMIC-IV, selecting tasks with documented disparities [sepsis mortality prediction showing racial bias (Amarasingham et al., 2010), readmission prediction showing socioeconomic gradients (Amarasingham et al., 2010)] and comparing audit findings to known clinical patterns. This would establish external validity beyond synthetic experiments.

*Single protected attribute and binary metrics*: We focused on gender with two fairness metrics. Real-world fairness audits must address intersectionality, disparities concentrated at intersections of multiple attributes where aggregate metrics mask severe localized biases. Methodological challenges include exponential subgroup growth and small-sample instability. Future work should develop subgroup discovery algorithms and hierarchical testing procedures to address these challenges.

*Fairness metric selection*: Numerous fairness definitions exist (demographic parity, equal opportunity, predictive parity, calibration, individual fairness), often mathematically conflicting. Our framework is metric-agnostic but does not resolve normative questions about which definition(s) should be prioritized in clinical contexts. Future research should develop decision frameworks for context-appropriate metric selection, incorporating patient and community perspectives through participatory design.

*From detection to mitigation*: Our framework detects and characterizes bias but does not automatically mitigate it. Future work should develop closed-loop pipelines integrating provenance audit with mitigation strategy selection, detecting bias, diagnosing mechanism through log analysis, recommending interventions, applying them, and validating improvement through re-audit.

*Model architecture scope*: We evaluated logistic regression and random forest. Modern clinical AI increasingly uses deep neural networks. Extending provenance to deep learning requires adaptation: gradient-based attribution methods (SHAP (Lundberg and Lee, 2017), Integrated Gradients (Sundararajan et al., 2017)), layer-wise provenance logging, and assessment of whether architectural complexity fundamentally limits interpretability.

*Deployment and organizational factors*: Computational experiments cannot substitute for real-world deployment studies. Implementing provenance auditing in a live hospital environment would reveal operational challenges (EHR integration, database infrastructure, query performance), user acceptance factors (clinician attitudes, administrative burden), and regulatory considerations (HIPAA compliance, legal defensibility) that laboratory studies cannot anticipate. We are developing a deployment protocol for a six-month pilot to address these questions.

## 5 Conclusion

AI has the potential to greatly enhance clinical decision-making, but issues of fairness and transparency cannot be an afterthought, they are fundamental to safe and effective care. In this paper, we presented a provenance-based simulation study and framework that operationalizes fairness auditing for clinical AI systems. By recording the “story” of each AI decision through provenance logs, we demonstrated how hidden biases can be brought to light and addressed. Our comparative analysis of logistic regression and random forest models underscored that even moderately accurate models can exhibit biased behavior, yet with the right audit tools, these behaviors become traceable and explainable. We introduced the concept of an *AI Fairness Provenance Record*, essentially a living document (via secure logs) of an AI’s development, performance, and decision rationale. This record serves as a foundation for accountability; it allows hospitals to answer the tough questions about their AI: *Is it fair? How do we know? What happened in this specific case?* With evidence rather than guesswork.

Our work contributes a concrete approach to meet emerging regulatory demands. We showed that our framework aligns closely with the U.S. FDA’s guidelines on transparency and the new ONC rule on algorithmic transparency (U.S. Food and Drug Administration, 2024; Dlapiper, 2023). As regulatory scrutiny of AI in healthcare intensifies, such alignment is not only beneficial but will become necessary. Importantly, the spirit of our approach is not merely about compliance; it is about fostering trust. Clinicians and patients will trust AI when they can see that it’s being monitored and that its advice can be verified and understood. Auditable AI, as we envision, behaves like a good clinician: it keeps thorough records, it can justify its recommendations with evidence, and it is willing to be second-guessed and learn from mistakes.

Building fair AI is not a one-time fix but a continuous commitment. Regular bias audits, stakeholder involvement, and agility in responding to audit findings are all part of robust AI governance. Our provenance framework is a tool to facilitate that governance, a means for technical teams to communicate with oversight teams through data, and for oversight teams to have concrete checkpoints on AI behavior.

In conclusion, ensuring fairness in clinical AI is a shared responsibility of developers, hospitals, regulators, and the AI systems themselves. Provenance-based auditing empowers AI systems to bear their share of that responsibility by making their behavior transparent and traceable. As several research including Alu (2023) and Buolamwini and Gebru (2018) work in bias and trust suggests, awareness and proactive management of bias is key to harnessing AI’s benefits without exacerbating disparities (Alu, 2023). By implementing the strategies outlined here, we can move closer to AI that is not only intelligent and accurate, but also equitable and worthy of the trust we place in it to guide life-affecting decisions.

## Data Availability

The original contributions presented in the study are included in the article. The synthetic dataset and analysis code used in this study are publicly available at: https://drive.google.com/drive/folders/1ltG5O7Z_6a4M9d_4m6dhhF1EyNDOvXHd?usp=sharing. Further inquiries can be directed to the corresponding author.
